# Single-Cell Ribonucleic Acid Sequencing Clarifies Cold Tolerance Mechanisms in the Pacific White Shrimp (*Litopenaeus Vannamei*)

**DOI:** 10.3389/fgene.2021.792172

**Published:** 2022-01-12

**Authors:** Weilin Zhu, Chunling Yang, Xiuli Chen, Qingyun Liu, Qiangyong Li, Min Peng, Huanling Wang, Xiaohan Chen, Qiong Yang, Zhenping Liao, Min Li, Chuanyan Pan, Pengfei Feng, Digang Zeng, Yongzhen Zhao

**Affiliations:** ^1^ Guangxi Key Laboratory of Aquatic Genetic Breeding and Healthy Aquaculture, Guangxi Academy of Fishery Sciences, Nanning, China; ^2^ Guangxi Shrimp and Crab Breeding Engineering Technology Research Center, Guangxi Academy of Fishery Sciences, Nanning, China; ^3^ Key Lab of Freshwater Animal Breeding, Key Laboratory of Agricultural Animal Genetics, Breeding and Reproduction, Ministry of Education, College of Fishery, Huazhong Agriculture University, Wuhan, China

**Keywords:** single-cell RNA sequencing, Litopenaeus vannamei, cold tolerance, mechanisms, pacific white shrimp

## Abstract

To characterize the cold tolerance mechanism of the Pacific white shrimp (*Litopenaeus vannamei*), we performed single-cell RNA sequencing (scRNA-seq) of ∼5185 hepatopancreas cells from cold-tolerant (Lv-T) and common (Lv-C) *L. vannamei* at preferred and low temperatures (28°C and 10°C, respectively). The cells fell into 10 clusters and 4 cell types: embryonic, resorptive, blister-like, and fibrillar. We identified differentially expressed genes between Lv-T and Lv-C, which were mainly associated with the terms “immune system,” “cytoskeleton,” “antioxidant system,” “digestive enzyme,” and “detoxification,” as well as the pathways “metabolic pathways of oxidative phosphorylation,” “metabolism of xenobiotics by cytochrome P450,” “chemical carcinogenesis,” “drug metabolism-cytochrome P450,” and “fatty acid metabolism.” Reconstruction of fibrillar cell trajectories showed that, under low temperature stress, hepatopancreas cells had two distinct fates, cell fate 1 and cell fate 2. Cell fate 1 was mainly involved in signal transduction and sensory organ development. Cell fate 2 was mainly involved in metabolic processes. This study preliminarily clarifies the molecular mechanisms underlying cold tolerance in *L. vannamei*, which will be useful for the breeding of shrimp with greater cold tolerance.

## Introduction

Temperature may directly affect shrimp metabolism, shelling, growth rate, oxygen consumption, and survival rate; temperature may also indirectly affect shrimp growth via environmental factors such as salinity and microalgae growth (Moullac and Haffner, 2000). Temperature also significantly affects prawn blood cell density (Gomez-Jimenez et al., 2000; Cheng and Chen, 2001), phagocytic activity (Steenbergen et al., 1978), and body fluid phenoloxidase activity. Indeed, cold is one of the main environmental factors affecting the growth and survival of the Pacific white shrimp (*Litopenaeus vannamei*): suitable growth temperatures for *L. vannamei* are 25–35°C, and this species poorly tolerates cold exposure. At temperatures below 18°C, *L. vannamei* stop eating (Ponce-Palafox et al., 1997). Low temperatures also limit *L. vannamei* breeding season duration and breeding range, affecting the economics of shrimp breeding. In order to breed *L. vannamei* with a greater range of tolerated temperatures, it is important to study the molecular genetic mechanisms underlying low temperature tolerance in this species. Although some genetic studies of cold tolerance in *L. vannamei* are available, these studies used high-throughput sequencing to identify microRNAs related to cold adaptation (He et al., 2018), to characterize the transcriptomes of the liver and pancreas under low temperature stress (Zhuo et al., 2021), and to investigate proteomic features (Peng et al., 2018; Fan et al., 2019).

Single-cell transcriptome sequencing is useful because even cells derived from the same individual or cell line may differ genomically, transcriptomically, and epigenetically (Wen and Tang, 2016) due to cellular heterogeneity (Kalisky et al., 2011). Conventional multi-cell transcriptomic analyses require large numbers of cells, and the results thus reflect average gene-expression values across the cell population used. As these average values mask differences in expression among cellular subgroups, it is difficult to distinguish the transcriptional characteristics of these subgroups within the cellular population (Lu and Fuchou, 2014; Wang and Navin, 2015). Single-cell transcriptome sequencing (scRNAseq) solves this problem by revealing expression differences among cell populations at the level of the individual cell (Gawad et al., 2016). In particular, scRNAseq can be used to isolate, identify, and describe different cell populations by sequencing the transcriptome libraries prepared from thousands of single cells in parallel (Trapnell, 2015; Moris et al., 2016; Tanay and Regev, 2017). Because scRNAseq analysis allows for the posterior identification of each cell in a cellular population, it is possible to study heterogeneous cell populations to discover unknown cell types or to compare different points in the developmental trajectory of a single cell or cell type (Briggs et al., 2018; Farrell and Wang, 2018; Wagner et al., 2018).

To date, single-cell transcriptome sequencing has rarely been applied to aquatic animals, and the few available studies have tended to focus on model fish or ascidians. For example, single-cell sequencing was used to reveal the heterogeneous effects of bisphenol A on zebrafish embryo development (Chen et al., 2020); to characterize the diversity of newborn neurons in the adult zebrafish brain (Lange and Rost, 2020); to show that homologous genes in human and zebrafish oocytes are highly expressed and belong to similar functional categories (Can et al., 2020); to reconstruct the trajectory of zebrafish embryogenesis and development (Farrell and Wang, 2018); to produce a transcriptome of zebrafish gene expression from the pharyngeal embryo stage to the larval stage (Farnsworth et al., 2020); to create a transcriptome map of the larval brain of *Ciona* (Sharma et al., 2019); to determine the regulatory network of dopaminergic neurons in embryonic ascidians (Horie et al., 2018); and to analyze head kidney-derived leukocytes in tilapia (Niu et al., 2020).

However, single-cell transcriptome sequencing has not previously been used to analyze the cold response of in *L. vannamei*. Here, we chose to focus on the hepatopancreas, as this organ plays an important role in immunity, hematopoiesis, metabolism, energy storage, detoxification, and excretion in shrimp (Yepiz-Plascencia et al., 2000; Verri et al., 2001; Shekhar et al., 2013). We therefore aimed to construct a single-cell map of the hepatopancreas of *L. vannamei* and to analyze the transcriptomes of its highly heterogeneous cell population; to compare singe-cell transcriptomes between cold-tolerant and common *L. vannamei*; and to characterize the cold tolerance mechanism of *L. vannamei*.

## Method Details

### Shrimp Used and Rapid Cooling Stress

Cold-tolerant *L. vannamei* (Lv-T) and common (Lv-C) *L. vannamei* were provided by Guangxi Shrimp Genetics and Breeding Center (Fangchenggang City, Guangxi Province, China). The average survival time (30.07 h) of Lv-T shrimp at 10°C are significantly greater than those (20.17 h) of Lv-C shrimp (Supplementary Table S1). All shrimp (average weight ∼32 g) were kept in the seawater pools (5 m × 3 m × 2 m) at a water temperature of 28 ± 0.5°C, salinity of 30‰, and pH of 7.9 ± 0.1 for 1 month to adapt to the experimental environment. After the acclimation period, an air-cooled condenser (FNV-73.9/240, Shengzhou Xinba Refrigeration Equipment Co., Ltd., Shaoxing City, Zhejiang Province, China) was used to reduce the water temperature, at a rate of 1°C per hour, from 28°C to 10°C and then to hold the water temperature at 10°C for 24 h. We randomly selected shrimp from among the cold-tolerant and common shrimp before cooling and after 24 h at 10°C to establish four treatment groups (*n* = 5 shrimp per group): cold-tolerant shrimp at 10°C (Lv-T10), cold-tolerant shrimp at 28°C (Lv-T28), common shrimp at 10°C (Lv-C10), and common shrimp at 28°C (Lv-C28). The hepatopancreas was removed from each selected shrimp, immediately transferred to MACS Tissue Storage Solution (Miltenyi Biotec, Germany), and held at 2–8°C for later use.

### Preparation of Hepatopancreas Single-Cell Suspensions

We cut 100 mg of each stored shrimp hepatopancreas sample into small pieces. We added the pieces to pre-cooled HBSS buffer (4°C) and gently pipetted the pieces in and out five times to wash the blood from the hepatopancreas. After natural sedimentation, the supernatant was discarded. Enzyme solution (2.5 g/L trypsin plus 0.1 g/L EDTA at a ratio of 1:1) was added to the clean tissue and allowed to digest for 10 min. When the tissue had swelled noticeably and appeared bleached, 2 ml of pre-cooled HBSS buffer solution was added and repeatedly pipetted gently to dissociate and release the hepatopancreas cells. After natural sedimentation for about 15 s, the cells were transferred to a new centrifuge tube using a pipette. This step was repeated three times for each sample (using the same centrifuge tube) to recover as many hepatopancreas cells as possible. The resulting cell suspension was filtered using a cell sieve with a pore size of 40 μm. The filtered liquid was centrifuged at 500 g/min for 3 minutes, the supernatant was discarded, and the remaining bottom layer (the cell pellet) was retained. Each cell pellet was resuspended in HBSS buffer, stained with trypan blue, and observed under a microscope to calculate cell concentration and percent viability. Cell suspension with cell viability >90% and cell concentration of 1,000–5000 cell/μL was used for future sequencing.

### cDNA Library Preparation and Single Cell RNA-Sequencing

Cell concentrations were adjusted to approximately 1,000 cell/μL using HBSS buffer. Gel beads containing barcode information were bound to the mixture of cells and enzymes, and bound molecules were then wrapped in oil surfactant droplets using the microfluidic “double cross” system to create gel beads-in-emulsions (GEMs). The GEMs flowed into the reservoir and were collected, the gel beads were dissolved to release the barcode sequences, and the cDNA fragments were reverse transcribed. The cDNA sequences were used as templates for PCR amplification, and the PCR amplicons were mixed to construct a sequencing library. The established libraries were sequenced using the Illumina sequencing platform.

### Cell Clustering

The raw sequences were cleaned by comparison to the *L. vannamei* genome (Zhang et al., 2019). Cell Ranger was used to filter, compare, quantify, identify, and reclaim cells; this software also generated a gene expression matrix for each cell. We then used Seurat to perform additional cell filtration and standardization, to classify cell clusters, to identify the genes differentially expressed among subgroups, and to screen marker genes. Cells that met the following conditions were retained for the future analysis: containing 400–1,000 genes; containing 750–3,000 unique molecular identifiers (UMI); and <20% of the UMIs were mitochondrial genes. The gene expression data for each retained cell were normalized against total gene expression levels across all retained cells using the global-scaling normalization method “LogNormalize” in the R software package Seurat 3.0 (https://satijalab.org/seurat/). Specifically, the gene expression data were multiplied by a scaling factor (10,000) and loge transformed as follows: gene expression level = ln (1 + (UMIA ÷ UMITotal) × 10,000), where UMIA was the UMI of gene A in the target cells; and UMITotal was the sum of all UMI quantities in the target cell. We performed a canonical correspondence analysis (CCA) of the normalized expression levels to eliminate the batch effect, and then integrated the expression data. The integrated data were Z-score normalized and a principal component analysis (PCA) was performed to reduce variable dimensionality. Finally, graph theory clustering algorithms were used to cluster cells across all four treatment groups. We then used t-SNE (McDavid et al., 2013) to visualize and explore the cell clusters.

### Differentially Expressed Transcripts and Functional Annotation

The likelihood-ratio test (Fonville et al., 2013) was used to identify patterns of differential gene expression among cell clusters. Significantly upregulated genes in each cluster were identified as those meeting all of the following criteria: expressed in >25% of all cells in the experimental (cold) treatment or in the control treatment, and log2FC was ≥0.360,674 (*p* ≤ 0.01) between the control and experimental treatment groups (i.e., upregulated at least 1.28-fold in response to cold). The five most strongly upregulated genes (i.e., with the greatest logFC values) in each cell cluster were selected as potential marker genes for that cluster. We then constructed heat maps and bubble charts for the marker genes.

We annotated all of the transcripts differentially expressed between the cold (experimental) treatment and the control treatment across all cell clusters using the GO database (http://www.geneontology.org/) (Falcon and Gentleman, 2007). We counted the number of transcripts associated with each term and performed a hypergeometric test to identify GO terms that were significantly enriched in the differentially expressed transcripts (FDR-corrected *p* ≤ 0.05). We determined the primary biological functions of each of the differentially expressed transcripts based on the significantly enriched GO functions. We then used the hypergeometric test, followed by correction for the false discovery rate (FDR), to identify pathways in the KEGG database (Kanehisa and Goto, 2000) that were significantly enriched in differentially expressed transcripts (Q-value ≤0.05). Based on the pathways significantly enriched in the differentially expressed transcripts, particularly those associated with biochemical metabolisms and signal transduction, we manually grouped the cell clusters into the four major cell types found in the crustacean hepatopancreas tubules: resorptive cells (R-cells), blister-like cells (B-cells), fibrillar cells (F-cells), and embryonic cells (E-cells).

### Differentially Expressed Gene Analysis

To explore molecular differences in the cold stress response between cold-tolerant (Lv-T) and common (Lv-C) *L. vannamei*, we performed scRNA-based transcriptome analyses on samples from the four treatment groups (Lv-T10, Lv-T28, Lv-C10, and Lv-C28). We identified DEGs in four sets of comparisons: Lv-C28-vs-Lv-C10 using Seurat (Stuart et al., 2019); genes were considered significantly differentially expressed when |log2FC| was ≥0.36, the proportion of cells expressing the target gene in a given cluster was ≥0.1, and ≥25% of all cells in that same cluster expressed the target gene. We then used the hurdle model in Model-based Analysis of Single-cell Transcriptomics (MAST) (Finak et al., 2015) to identify the DEGs, based on each set of comparisons, in each cluster. *p*-values were corrected for multiple tests using the Benjamini-Hochberg method in Seurat; and genes were considered DEGs when the corrected *p*-value was ≤0.05. We then analyzed the GO and KEGG enrichment of the DEGs as described in Section 2.5.

### Pseudotime Analysis

Single-cell gene expression profiles allow the deconstruction of population heterogeneity and the reconstruction of cell differentiation trajectories. As F-cells are important for hepatopancreatic function in *L. vannamei* cells, we chose to investigate the differentiation trajectory of these cells in response to cold stress using pseudotime (cell-cell trajectory) analysis (Trapnell et al., 2014) in Monocle (Tryselius and Hultmark, 1997; Qiu et al., 2017). In brief, we used Monocle to reduce the single-cell gene expression data matrix to two dimensions. The cells were plotted as a minimum spanning tree, and difference in expression were determined based on biological meaning. We considered the original cell cluster that with the lowest pseudotime in the cell trajectory, and the pseudotime values for all cells were calculated from this. Based on pseudotime values, Monocle models gene expression levels as smooth, non-linear pseudotime functions to visualize changes in gene expression level over pseudotime. Genes with FDR<1e-5 were identified as differentially expressed genes over pseudotime.

## Results

### Ten Cell Clusters Were Identified Across 5185 Cells From the Four Treatment Groups

**FIGURE 1:**
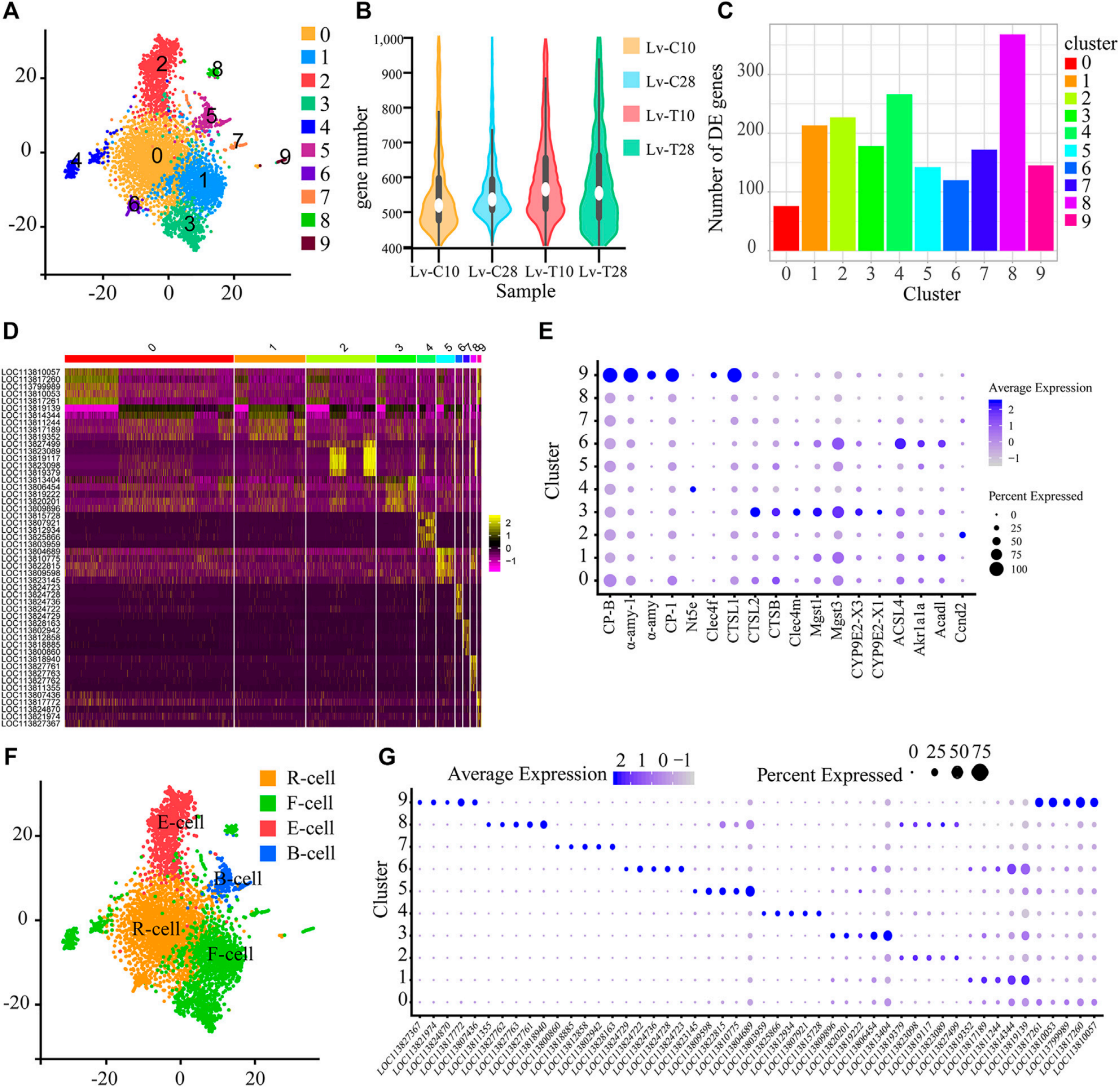
Single-cell sequencing reveals the subtypes of shrimp hepatopancreatic cells. **(A)** t-SNE visualization showing the 10 single-cell clusters identified in the 5185 hepatopancreas cells. Each dot represents a single cell; clusters are distinguished by color. **(B)** The number of genes per cell in each of the four treatments. **(C)** Histogram showing the number of genes upregulated in the cold (experimental) treatment group as compared to the control group in each of the single-cell subgroups shown in panel **(A)**. **(D)** Heat map showing the expression patterns of the five most strongly upregulated genes in each cell cluster. Each column represents a cell, and each row represents a gene. Brighter yellow cells are more upregulated; brighter pink cells are more downregulated. **(E)** Bubble chart showing the expression levels of candidate molecular marker genes across all cell subgroups. The size of the bubble represents the ratio of the total expression of each marker gene in the corresponding cluster to its total expression across all clusters. That is, larger bubbles indicate genes that were relatively frequently expressed in the corresponding cluster, while darker bubbles indicate genes that were relatively highly expressed in the corresponding cluster. **(F)** t-SNE visualization showing the cell clusters classified into the four major types of hepatopancreas cells: resorptive cells (R-cells; orange), blister-like cells (B-cells; blue), fibrillar cells (F-cells; green), and embryonic cells (E-cells; red). Cell clusters were classified as cell types based on the molecular markers and KEGG signaling pathways. **(G)** Bubble chart showing the expression levels of candidate molecular marker genes across all cell subgroups. The size of the bubble represents the ratio of the total expression of each marker gene in the corresponding cluster to its total expression across all clusters. That is, larger bubbles indicate genes that were relatively frequently expressed in the corresponding cluster, while darker bubbles indicate genes that were relatively highly expressed in the corresponding cluster.

Illumina sequencing identified 61,193 genes in 252,817 *L. vannamei* hepatopancreas cells across the four treatment groups: cold-tolerant shrimp at 10°C (Lv-T10), cold-tolerant shrimp at 28°C (Lv-T28), common shrimp at 10°C (Lv-C10); and common shrimp at 28°C (Lv-C28). Of these cells, 5185 hepatopancreas cells were identified as high quality, based on the number of genes per cell (100–400; Figure 1B), the number of UMIs per cell (750–3,000), and the proportion of UMIs that were mitochondrial genes (<20%). These 5185 cells clustered into 10 subgroups (Figure 1A) using Seurat, a clustering algorithm based on graph theory.

To analyze the transcriptional regulation of each cell cluster, and to screen marker genes unique to each cluster, we first identified the genes in each cluster that were significantly upregulated in response to cold stress. In cell clusters 0–9, we identified 76, 213, 227, 178, 266, 142, 120, 172, 368, and 145 significantly upregulated genes, respectively, in the cold treatment group as compared to the control treatment group (Figure 1C). According to the expression analysis of genes in each cluster, we further selected the five most strongly upregulated genes in each cluster as marker genes. A heatmap (Figure 1D) shows the expression patterns of the marker genes, and an enriched bubble chart (Figure 1E) and a bubble chart (Figure 1G) show the expression levels of marker genes across all cell subgroups. These genes may be useful molecular markers for these cell clusters in future studies.

### The 10 Cell Clusters Were Classified Into Four Cell Types

According to pathways and molecular markers, we classified the 10 cell clusters into 4 cell types, including R-cells, B-cells, F-cells, and E-cells (Figure 1F) as follows (Niu et al., 2020): Several pathways related to the fat metabolism, including “fatty acid metabolism” (ko01212), “fatty acid degradation” (ko00071), and the “glyoxylate and dicarboxylate metabolism” (ko00630), were significantly enriched in cell cluster 0; in particular, the glyoxylate and dicarboxylate metabolism is important for the conversion of fat to glycogen. Two molecular function GO terms associated with metal ions, “metalloexopeptidase activity” (GO:0,008,235) and “metallopeptidase activity” (GO:0,008,237), were also significantly enriched in cell cluster 0. As R-cells primarily absorb and store nutrients (e.g., lipid droplets, glycogen, and calcium) and trace elements (e.g., copper, zinc, sulfur, and calcium) (Al-Mohanna and Nott, 1987), but are also involved in fat metabolism (Sousa and Petriella, 2001; Tian et al., 2012), we classified cell cluster 0 as R-cells (Figure 1F). The pathway “glycosphingolipid biosynthesis-ganglio series” (ko00604) was significantly enriched in cell subgroup 6. Glycosphingolipids are complex glycolipids (Chatterjee and Kwiterovich, 1984). As lipid storage is an important function of R-cells (Sousa and Petriella, 2001; Tian et al., 2012), we also classified cell cluster 6 as R-cells (Figure 1F).

Several pathways and GO cellular component terms associated with B-cells, including “phagosome” (ko04145), “endocytosis” (ko04144), “lysosome” (ko04142), “synaptic vesicle cycle” (ko04721), “lysosome” (GO:0,005,764), “secondary lysosome” (GO:0,005,767), “vacuum lumen” (GO:0,005,775), and “autophagosome” (GO:0,005,776) were significantly enriched in cell cluster 5. As mature B-cells contain large amounts of rough endoplasmic reticulum (rER), Golgi apparatus, mitochondria, and lysosomes, and exhibit endocytosis (Vogt, 2019), we classified cell cluster 5 as B-cells (Figure 1F).

The “steroid hormone biosynthesis” (ko00140) pathway was significantly enriched in cell cluster 4. The synthesis of steroid hormones is controlled by several highly selective cytochrome P450 enzymes, in conjunction with some steroid dehydrogenases and reductases, especially aromatase (Sanderson, 2006). In addition, the hepatopancreas detoxifies organic compounds using cytochrome P450 (James and Boyle, 1998; Brouwer and Lee, 2007). We also identified 189 transcripts associated with detoxification in cell clusters 1 and 4. These transcripts were significantly enriched in the pathways “drug metabolism-cytochrome P450” (ko00982), “metabolism of xenobiotics by cytochrome P450” (ko00980), and “longevity regulating pathway-worm” (ko04212) in cell clusters 1 and 4. In cell cluster 1, the “glutathione S-transferase genes” pathway (including the three subtypes LOC113817634, LOC113822637, and LOC113829084) was significantly enriched in the “longevity regulating pathway-worm” (ko04212) pathway. There is evidence that the “glutathione S-transferase gene” is localized in the F-cells of the crab *Callinectes sapidus*, and this enzyme plays an important role in the detoxification of organic heterologous substances (Keeran and Lee, 1987). Indeed, in several crustaceans the F-cells have been identified as the possible detoxification site for organic foreign bodies (Brignac-Huber et al., 2016; Vogt, 2019). We thus classified cell clusters 1 and 4 as F-cells (Figure 1F).

The “carbohydrate digestion and absorption” (ko04973) and “starch and sucrose metabolism” (ko00500) pathways were significantly enriched in cell cluster 8, while genes related to digestive enzymes, including carboxypeptidase B (CP-B), amylase (α-AMY), and cathepsin 1 (CTSL1), were significantly upregulated in cell subset 9. CTSL1, as well as CTSB, CLEC4M, Mgst1, Mgst3, CYP9E2, and CYP9E2 (Figure 1E), were also upregulated in cell cluster 3. CTSL1 is a typical lysosomal enzyme that is found in the digestive juices of decapod animals (Hu and Leung, 2007). in addition, previous *in situ* hybridization studies based on chymotrypsin (Van wormhoudt et al., 1995), amylase, chitinase, cellulase, and trypsin (Lehnert and Johnson, 2002) showed that F-cells were the main site of digestive enzyme synthesis. F-cells also synthesize digestive enzymes and blood proteins, and participate in oxygen transport and immune defense (Vogt et al., 1989; Möhrlen et al., 2001; Vogt, 2019). Finally, previous studies have shown that F-cells in *Penaeus penicillatus* secrete zymogen granules through exocytosis (Al-Mohanna et al., 1985). Thus, as the main function of F-cells is to synthesize and secrete digestive enzymes (Al-Mohanna et al., 1985; Toullec et al., 1992), we classified cell clusters 3, 8, and 9 as F-cells (Figure 1F).

Several signaling pathways related to immunity, including “B cell receptor signaling pathway” (ko04662), “viral carcinogenesis” (ko05203), “human cytomegalovirus infection” (ko05163), and “HTLV-I infection” (ko05166) were significantly enriched in cluster 7, while “B cell receptor signaling pathway” (ko04662), “human cytomegalovirus infection” (ko05163), “kaposi sarcoma-associated herpesvirus infection” (ko05167), “pathways in cancer” (ko05200), “leukocyte transendothelial migration” (ko04670), and “cell adhesion molecules (CAMs)” (ko04514) were significantly enriched in cluster 9. The most abundant protein in hemolymph, while plays a key role in the crustacean immune system, is the oxygen-carrier hemocyanin (>90% of all proteins in hemolymph) (Stöcker et al., 1988). As a previous study used *in situ* hybridization of shrimp hepatopancreas slices with hemocyanin to show that hemocyanin was synthesized in F-cells (Lehnert and Johnson, 2002; Wang et al., 2007), we classified the cell clusters 7 and 9 as F-cells.

The “hedgehog signaling pathway” (ko04340), which regulates cell fate, including proliferation, apoptosis, migration, and differentiation (Qu et al., 2021; Wutka et al., 2014), was significantly enriched in cell cluster 2. Ccnd2, which was significantly upregulated in cell cluster 2, is a key gene in the “hedgehog signaling” pathway (Li et al., 2020) (Figure 1E). E-cells are undifferentiated embryonic cells from which R-, B-, and F-cells are differentiated (Toullec et al., 1992; Sousa and Petriella, 2000; Tian et al., 2012). Therefore, we identified cell cluster 2 as E-cells.

### Differentially Expressed Genes Associated With Cold Tolerance

**FIGURE 2:**
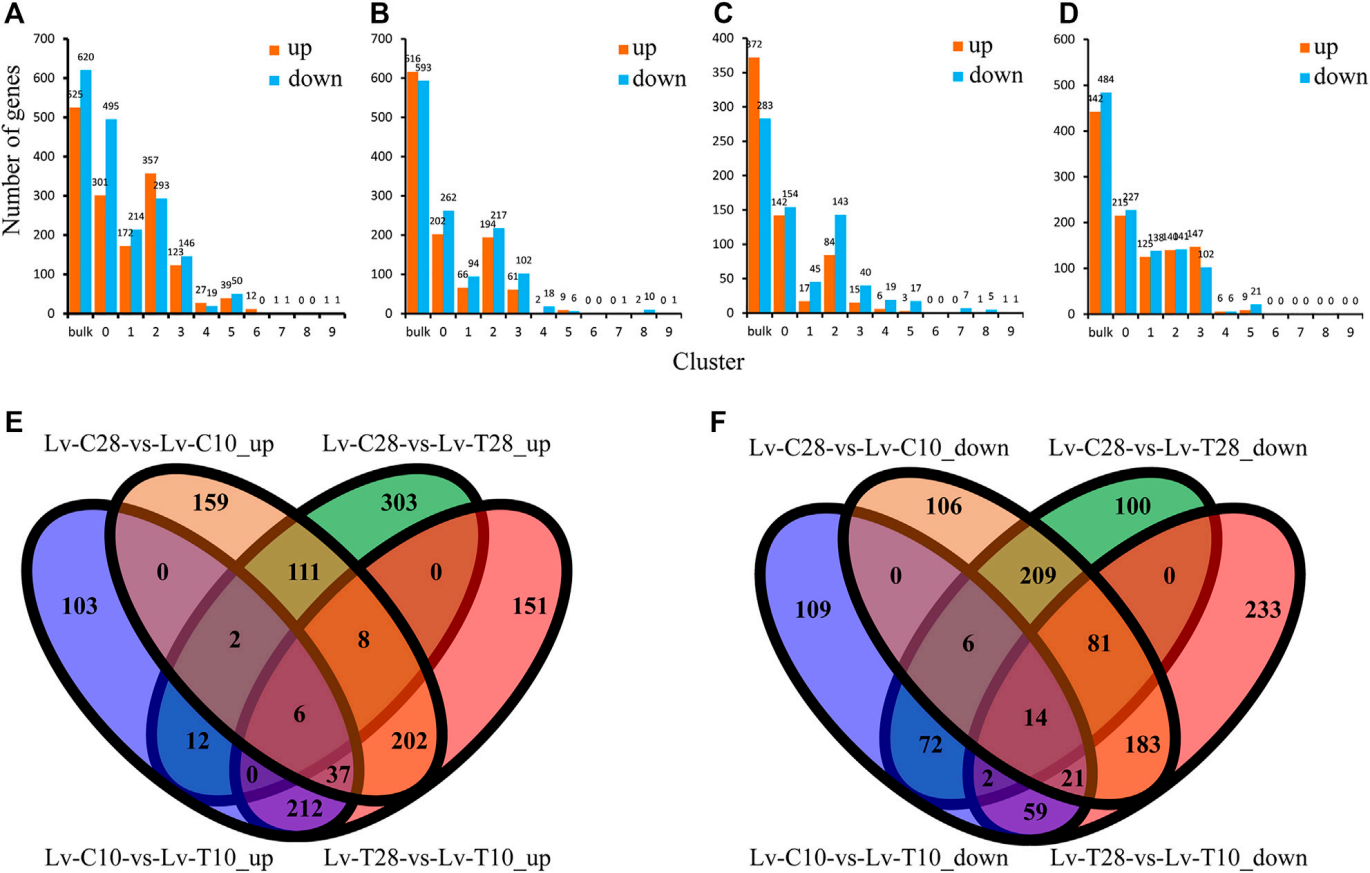
Differentially expressed genes (DEGs) between cold-tolerant and common *Litopenaeus vannamei* at 10°C and 28°C. **(A–D)** Differentially expressed genes across all cell cultures (Total) and in cell clusters 0–9 for the following comparisons: **(A)** Lv-C28-vs-Lv-C10 (common at 28°C versus common at 10°C), **(B)** Lv-T28-vs-Lv-T10 (cold-tolerant at 28°C versus cold-tolerant at 10°C), **(C)** Lv-C10-vs-Lv-T10 (common at 10°C versus cold-tolerant at 10°C), and **(D)** Lv-C28-vs-Lv-T28 (common at 28°C versus cold-tolerant at 28°C). **(E,F)** Venn diagrams showing the **(E)** upregulated and **(F)** downregulated genes shared and unique among the four comparisons.

Across all cell clusters, we identified 1,145 DEGs between common *L. vannamei* at 28°C and common *L. vannamei* at 10°C (Lv-C28-vs-Lv-C10), 525 upregulated and 620 downregulated (Figure 2A); 1,209 DEGs between cold-tolerant *L. vannamei* at 28°C and cold-tolerant *L. vannamei* at 10°C (Lv-T28-vs-Lv-T10), 616 upregulated and 593 downregulated (Figure 2B); 655 DEGs between cold-tolerant *L. vannamei* at 10°C and common *L. vannamei* at 10°C (Lv-C10-vs-Lv-T10), 372 upregulated and 283 downregulated (Figure 2C); and 926 DEGs between common *L. vannamei* at 28°C and cold-tolerant *L. vannamei* at 28°C (Lv-C28-vs-Lv-C28), 442 upregulated and 484 downregulated (Figure 2D).

Six genes were co-upregulated in all four comparison groups and 14 were co-downregulated (Figures 2E,F). The shared upregulated genes included Kintoun (LOC113828196), CtBP (LOC113800533), Hnrnph1 (LOC113819995), and La (LOC113809655), while the shared downregulated genes included nrf-6 (LOC113816674), PHYH (LOC113811729), AK (LOC113816366), and Gs2 (LOC113823144). We identified 202 co-upregulated DEGs (Figure 2E) and 183 co-downregulated DEGs (Figure 2F) in cold-tolerant and common shrimp at 28°C as compared to 10°C (i.e., Lv-C28-vs-Lv-C10 and Lv-T28-vs-Lv-T10), including ACBP (LOC113815675), Leng8 (LOC113805896), GAT-2 (LOC113816986), Ankrd2 (LOC113828833), and ND1 (LOC113820286).

NR annotations indicated that, across all four comparisons, many of the DEGs were related to immune defense, the cytoskeleton, the antioxidant system, digestive enzymes, and detoxification, including C-type lectin (CTL), leukocyte receptor (Lr8), hemocyanin (HC), integrin beta-PS-like (ITGB), actin (ACT), titin-like (Ttn), catalase (CAT), glutathione S-transferase (GST), glutathioneperoxidase (GPX), peroxiredoxin 4 (PRDX4), preamylase 1 (Amy1), glutamate carboxypeptidase (GCP), chymotrypsin BII (Prss2), UDP-glucuronosyltransferases (UGTs), cytochrome P450 (CYP450) (Table 1). Other DEGs included low-density lipoprotein receptor 2 (LDLR2), calcium-activated 4A-like (Clca4), taurine transporter (TAUT), heat shock protein (HSC70), zinc proteinase (Zinc), and vital membrane outer layer protein 1-like (VMP1) (Table 1).

**TABLE 1:** Genes differentially expressed in the hepatopancreas of *Litopenaeus vannamei* in response to cold stress. (N/A, not significantly differentially expressed).

Genes	Lv-C28-vs-Lv-T28	Lv-C10-vs-Lv-T10	Lv-C28-vs-Lv-C10	Lv-T28-vs-Lv-T10
Immune system				
C-type lectin (CTL)	UP	DOWN	UP	UP
leukocyte receptor cluster member 8 (Lr8)	UP	UP	N/A	N/A
chitin binding-like protein (CBP)	DOWN	UP	N/A	UP
Chitinase precursor (Chia)	DOWN	DOWN	UP	UP
hemocyanin (HC)	UP	UP	N/A	UP
integrin beta-PS-like (ITGB)	N/A	UP	DOWN	N/A
macrophage mannose receptor 1-like (MMR1)	UP	UP	UP	UP
**Cytoskeleton**				
Actin (ACT)	N/A	N/A	UP	DOWN
tropomyosin (TPM)	N/A	UP	UP	UP
flotillin-1 (FLOT1)	N/A	UP	UP	UP
titin-like (Ttn)	N/A	UP	UP	UP
**Antioxidant system**				
catalase (CAT)	N/A	N/A	DOWN	DOWN
glutathione S-transferase (GST)	UP	DOWN	DOWN	DOWN
glutathione peroxidase (GPX)	UP	N/A	N/A	N/A
peroxiredoxin 4 (PRDX4)	UP	UP	DOWN	DOWN
**Digestive enzyme system**				
preamylase 1 (Amy1)	DOWN	DOWN	UP	N/A
glutamate carboxypeptidase (GCP)	UP	N/A	N/A	DOWN
chymotrypsin BII (Prss2)	DOWN	DOWN	UP	UP
glucose transporters (GLUT)	N/A	up	N/A	UP
**Detoxification**				
UDP-glucuronosyltransferase (GST)	UP	N/A	DOWN	DOWN
UDP-glucuronosyltransferases (UGTs)	N/A	UP	DOWN	N/A
cytochrome P450 (CYP450)	UP	N/A	DOWN	DOWN
epoxide hydrolase 4-like (EPHX4)	UP	N/A	DOWN	DOWN
**Others**				
low-density lipoprotein receptor 2 (LDLR2)	N/A	N/A	UP	UP
calcium-activated 4A-like (Clca4)	DOWN	UP	DOWN	N/A
taurine transporter (TAUT)	UP	UP	N/A	N/A
heat shock protein (HSC70)	N/A	N/A	UP	UP
zinc proteinase (Zinc)	DOWN	DOWN	UP	UP
vitelline membrane outer layer protein 1-like (VMP1)	N/A	DOWN	UP	UP

### Pathways Significantly Enriched in the Differentially Expressed Genes Were Associated With Cold-Stress Resistance

**FIGURE 3:**
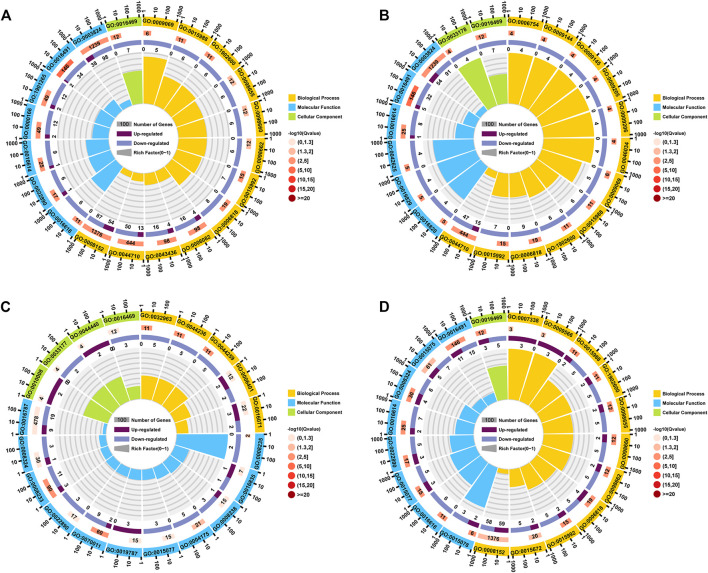
Circle plots showing GO enrichment of the genes differentially expressed between cold-tolerant and common *Litopenaeus vannamei* at 10°C and 28°C. **(A)** Lv-C28-vs-Lv-C10 (common at 28°C versus common at 10°C), **(B)** Lv-T28-vs-Lv-T10 (cold-tolerant at 28°C versus cold-tolerant at 10°C), **(C)** Lv-C10-vs-Lv-T10 (common at 10°C versus cold-tolerant at 10°C), and **(D)** Lv-C28-vs-Lv-T28 (common at 28°C versus cold-tolerant at 28°C). The outermost circle of each graph shows the 20 GO terms most commonly significantly enriched in the differentially expressed genes (DEGs). The second circle shows the number of genes associated with each GO term; redder bars correspond to greater Q-values. The third circle shows the ratio of upregulated to downregulated DEGs associated with each GO term as bar graph, with upregulated genes shown in light purple and downregulated genes shown in dark purple; the specific numbers of up- and downregulated genes are shown below bar. The innermost (fourth) circle shows the Rich Factor value for each GO term (i.e., the number of DEGs versus the number of non-DEGs associated with the GO term); each gridline represents 0.1.

The DEGs in the Lv-C28-vs-Lv-C10 dataset were most commonly significantly enriched in the GO biological process terms “metabolic process (GO:0,008,152)”, “single-organism metabolic process” (GO:0,044,710), and “oxoacid metabolic process” (GO:0,043,436); in the GO cellular component term “proton-transporting two-sector atpase complex” (GO:0,016,469); and in the GO molecular function terms “catalytic activity” (GO:0,003,824) and “oxidoreductase activity” (GO:0,016,491; Figure 3A). The DEGs in the Lv-T28-vs-Lv-T10 dataset were most commonly significantly enriched in the GO biological process terms “single-organism metabolic process” (GO:0,044,710), “proton transport (GO:0,015,992), and “hydrogen transport” (GO:0,006,818); in the GO cellular component terms “proton-transporting two-sector atpase complex” (GO:0,016,469) and “proton-transporting two-sector atpase complex, catalytic domain” (GO:0,033,178); and in the GO molecular function terms “catalytic activity” (GO:0,003,824), “oxidoreductase activity” (GO:0,016,491), and “oxidoreductase activity, acting on CH-OH group of donor” (GO:0,016,614; Figure 3B). The DEGs in the Lv-C10-vs-Lv-T10 dataset were most commonly significantly enriched in the GO biological process terms “mRNA metabolic process” (GO:0,016,071), “RNA catabolic process” (GO:0,006,401), and “multicellular organismal macromolecule metabolic process” (GO:0,044,259); in the GO cellular component terms “proton-transporting two-sector atpase complex” (GO:0,016,469) and “endosomal part” (GO:0,044,440); and in the GO molecular function terms “hydrolase activity” (GO:0,016,787), “cation transmembrane transporter activity” (GO:0,008,324), and “peptidase activity” (GO:0,008,233; Figure 3C). The DEGs in the Lv-C28-vs-Lv-T28 dataset were most commonly significantly enriched in the GO biological process terms “metabolic process” (GO:0,008,152), “monovalent inorganic cation transport” (GO:0,015,672), and “proton transport” (GO:0,015,992); in the GO cellular component term “proton-transporting two-sector atpase complex” (GO:0,016,469); and in the GO molecular function terms “oxidoreductase activity” (GO:0,016,491), “ion transmembrane transporter activity” (GO:0,015,075), and “cation transmembrane transporter activity” (GO:0,008,324; Figure 3D).

**FIGURE 4:**
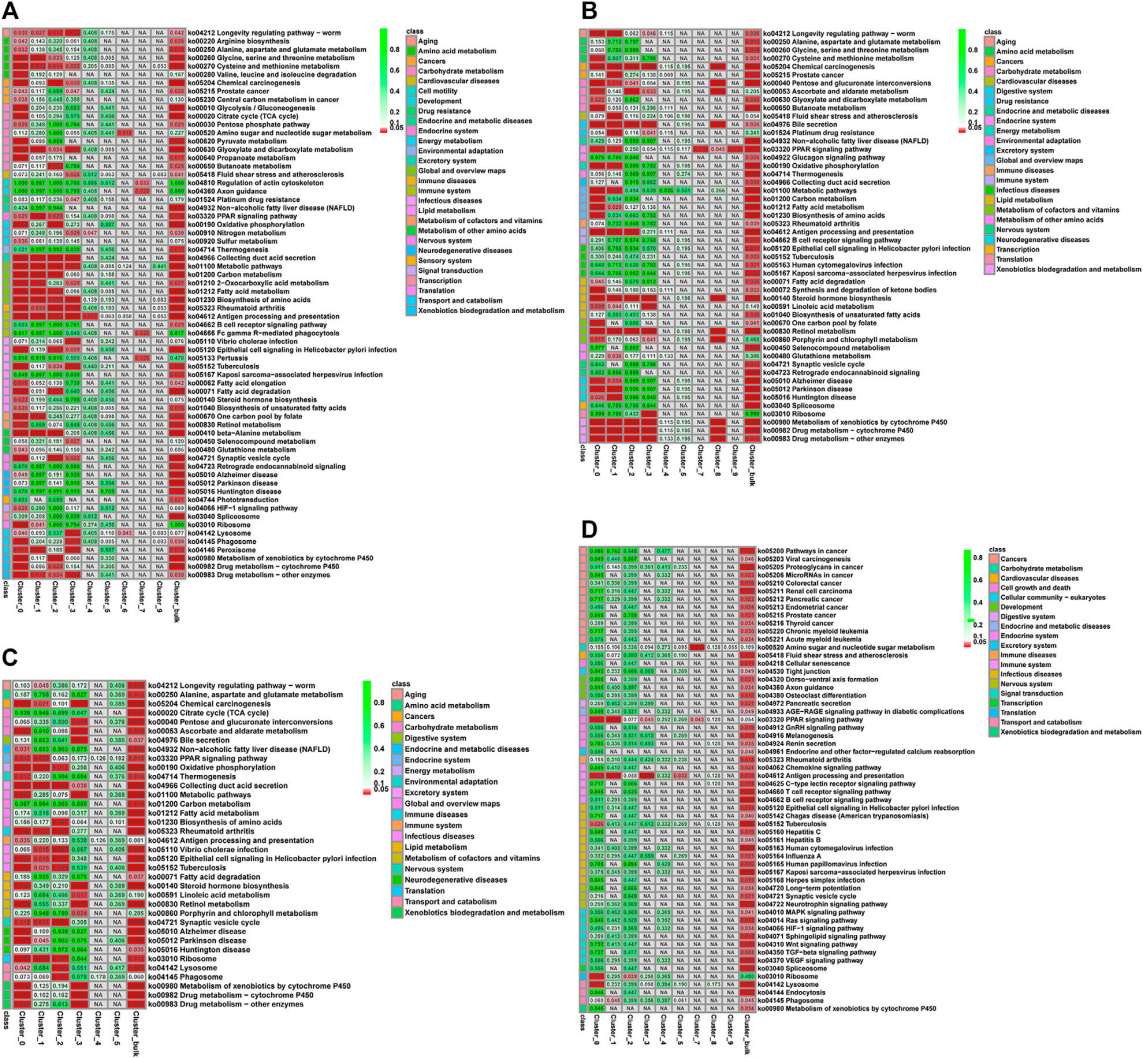
Q-value heatmaps of KEGG pathways associated with the genes differentially expressed between cold-tolerant and common *Litopenaeus vannamei* at 10°C and 28°C across all cell clusters (Total) and in individual cell clusters (0–9). **(A)** common at 28°C versus common at 10°C, **(B)** cold-tolerant at 28°C versus cold-tolerant at 10°C, **(C)** common at 10°C versus cold-tolerant at 10°C, and **(D)** common at 28°C versus cold-tolerant at 28°C. Q-values (i.e., *p*-values corrected for the false discovery rate) are shown in cells: cells where Q ≤ 0.05 are shown in red; cells where Q > 0.05 are shown in green; cells not corresponding to differential expression are shown in grey. Corresponding KEGG signaling pathways are shown in the leftmost column of each graph.

Nine KEGG pathways were significantly enriched (Q-value ≤ Q-val in the Lv-C28-vs-Lv-C10, Lv-T28-vs-Lv-T10, and Lv-C28-vs-Lv-T28 DEG datasets, including “oxidative phosphorylation” (ko00190), “metabolic pathways” (ko01100), “biosynthesis of amino acids” (ko01230), “metabolism of xenobiotics by cytochrome P450 " (Ko00980), “chemical carcinogenesis” (ko05204), “drug metabolism-cytochrome P450” (ko00982), and “fatty acid metabolism” (ko01212; Figure 4). These pathways may be related to cold stress. The pathways “pentose and glucuronate interconversions” (ko00040), “steroid hormone biosynthesis” (ko00140), and “bile secretion” (Ko04976) were significantly enriched in the Lv-C10-vs-Lv-T10 and Lv-C28-vs-Lv-T28 DEG datasets after cold stress, but not in the Lv-C28-vs-Lv-C10 DEG dataset. That is, these pathways were significantly enriched in the cold-tolerant shrimp, but not the common shrimp, in response to cold stress. Thus, these pathways may be important for cold-stress resistance in cold-tolerant *L. vannamei* families. Notably, these three pathways were also significantly enriched in cell cluster 0 (i.e., R-cells; Figure 4). In the DEG datasets Lv-C10-vs-Lv-T10 and Lv-C28-vs-Lv-T28, R-cells were significantly enriched in the “metabolism of xenobiotics by cytochrome P450” (ko00980), “chemical carcinogenesis” (ko05204), “drug metabolism-cytochrome P450” (ko00982), “antigen processing and presentation” (ko04612), and “drug metabolism-other enzymes” (ko00983) pathways (Figure 4).

Across all DEG datasets, R-cells were significantly enriched in the “antigen processing and presentation pathway” (ko04612) and “PPAR signaling pathway” (ko03320), while F-cells were significantly enriched the “PPAR signaling pathway” (ko03320). B-cells were not significantly enriched in any KEGG pathways in any DEG dataset. was significantly enriched in all comparison groups.

### F-Cell Trajectories Differed Between Cold-Tolerant and Common F-Cells in Response to Cold Stress

**FIGURE 5:**
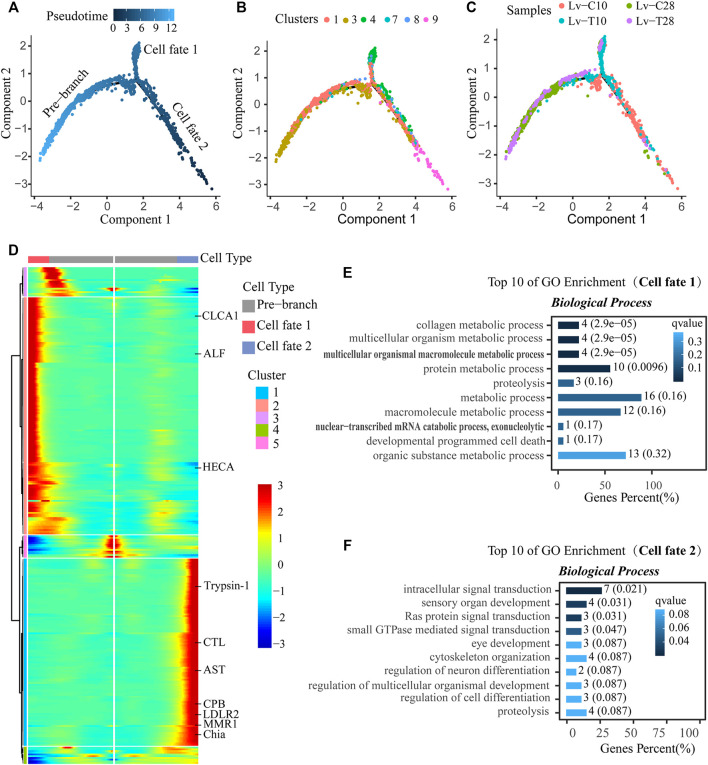
Reconstruction of the differentiation trajectory of F-cells in a quasi-chronological analysis. **(A–C)** Differentiation trajectory of F-cells with different mappings: **(A)** pseudotime, **(B)** cell clusters, and **(C)** treatment groups. Three cell trajectories are shown in these graphs: the pre-branch, cell fate 1, and cell fate 2. **(D)** Heatmap showing simulated gene expression dynamics in F-cells during cell state transitions from the pre-branch to cell fate 1 or 2. Pseudotime is shown on the *x*-axis (with the origin at the center of the axis), and genes are graphed along the *y*-axis. Gene sets with similar expression patterns are clustered. **(E,F)** The top 10 GO terms with the smallest Q value were used for mapping. GO terms are shown on the *y*-axis; the percentages of the number of GO terms to the total number of differences are shown on the *x*-axis (the darker the color, the smaller the Q value). The digitals on the columns are the GO term numbers and Q values. E is the branch of cell fate 1, and F is the branch of cell fate 2.

After F-cell differentiation in response to cold-stress (the pre-branch), 2 cell fates were identified: cell fate 1 and cell fate 2 (Figure 5A). F-cells from clusters 1 and 3 were primarily found at the beginning of the pre-branch, transitioning to cell clusters 7 and 8 just before differentiation. After differentiation, cell fate 1 primarily included cell cluster 4, while cell fate 2 primarily included cell cluster 9 (Figure 5B). The pre-branch primarily included cells from both common and cold-tolerant shrimp at 28°C; cells from the common shrimp at 10°C were mainly found on the cell fate 1 branch, while cells from the cold-tolerant shrimp at 10°C were mainly found on the cell fate 2 branch (Figure 5C).

**FIGURE 6:**
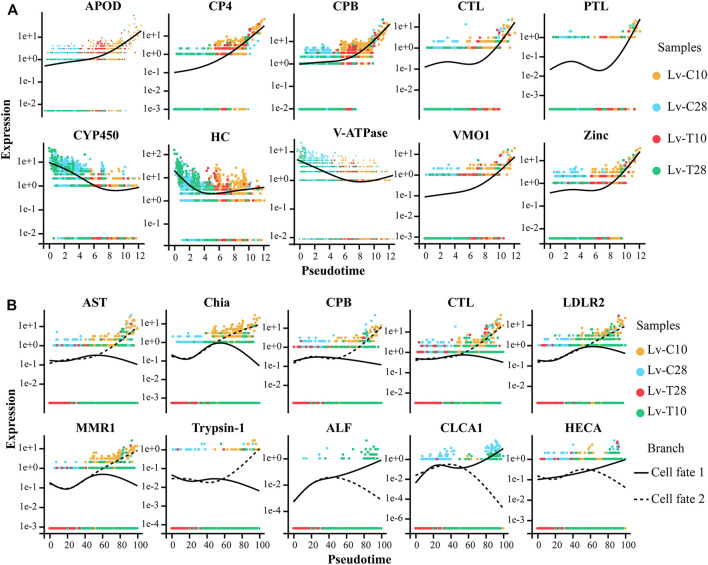
Expression patterns of genes in cold-tolerant and wild-type *Litopenaeus vannamei* in response to cold stress over pseudo-time. Yellow dot represents common at 10°C, blue dot represents common at 28°C, red dot represents cold-tolerant at 10°C, and green dot represents cold-tolerant at 28°C. **(A)** Gene expression patterns in cells with similar fates after cold stress. Solid black line represents best fit line for gene expression level. **(B)** Gene expression patterns in cells with different fates after cold stress. Solid black line represents the best fit line for the expression levels of genes in cells associated with cell fate 1, and the dashed line represents the best fit line for the expression levels of genes in cells associated with cell fate 2.

A heatmap showing gene expression dynamics over pseudotime showed distinct patterns of gene expression among the three branches (Figure 5D). Several genes exhibited similar expression patterns and cell fates over pseudotime in both common and cold-tolerant shrimp. For example, apolipoprotein D (APOD), cysteine proteinase 4-like (CP4), carboxypeptidase B (CPB), pancreatic triacylglycerol lipase-like (PTL), vitelline membrane outer Layer 1 (VMO1), and zinc proteinase (Zinc) were upregulated, while cytochrome P450 9e2-like (CYP450), hemocyanin (HC), and V-type proton atpase (V-ATPase) were downregulated (Figure 6A). Other genes exhibited different expression patterns in cells with different fates in response to cold stress. For example, astacin-like (AST), chitinase precursor (Chia), carboxypeptidase B (CPB), C-type lectin receptor (CTL), low-density lipoprotein receptor-related protein 2-like (LDLR2), macrophage mannose receptor 1-like (MMR1), and trypsin-1-like (Trypsin-1) were upregulated in cells on the cell fate 2 branch, but downregulated in cells on the cell fate 1 branch (Figure 6B). Conversely, anti-lipopolysaccharide factor (ALF), calcium-activated chloride channel regulator 1 (CLCA1), and headcase protein-like (HECA) were downregulated in cells on the cell fate 2 branch, but upregulated in cells on the cell fate 1 branch (Figure 6B).

GO enrichment analysis indicated that cell fate 1 was mainly associated with signal pathway transduction and sensory organ development: the GO terms “intracellular signal transduction" (GO:0,035,556), “sensory organ development" (GO:0,007,423), “Ras protein signal transduction" (GO:0,007,265), and “small gtpase mediated signal transduction" (GO:0,007,264) were significantly enriched in cell fate 1 (Figure 5E). Cell fate 2 was mainly associated with metabolic processes: “collagen metabolic process" (GO:0,032,963), “multicellular organism metabolic process" (GO:0,044,236), “multicellular organismal macromolecule metabolic process" (GO:0,044,259), and “protein metabolic process" (GO:0,019,538) were significantly enriched in cell fate 2 (Figure 5F). Thus, pseudotime analysis showed that F-cells had different trajectories in cold-tolerant and common *L. vannamei* in response to low-temperature stress.

## Discussion

### Cell Clusters Identification in the Hepatopancreas of *L. Vannamei*

Although electron microscopic observation of hepatopancreas tubules have shown that the crustacean hepatopancreas is primarily composed E-cells, R-cells, B-cells, and F-cells (Al-Mohanna and Nott, 1989; Toullec et al., 1992; Sousa and Petriella, 2000; Hopkin and Nott, 2009), precise molecular markers for these cells types have yet to be identified. To address this knowledge gap, we comprehensively analyzed gene expression in the shrimp hepatopancreas at the level of a single cell, using scRNA-seq to identify candidate markers for each cell type in the shrimp hepatopancreas.

R-cells play important roles in the absorption and metabolism of nutrients, in energy (lipids and glycogen) and mineral element storage, in lipoprotein synthesis and export, in heavy metal detoxification, and in uric acid excretion (Al-Mohanna and Nott, 1987; Sousa and Petriella, 2001; Tian et al., 2012). Here, the pathways significantly enriched in the putative R-cells, including “glyoxylate and dicarboxylate metabolism,” “cysteine and methionine metabolism,” “metalloexopeptidase activity,” “metalloexopeptidase activity,” and “glycosphingolipid biosynthesis-ganglio series” were consistent with the functions of R-cells. The pathways significantly enriched in the putative F-cells, including “drug metabolism-cytochrome P450” and “metabolism of xenobiology by cytochrome P450” were consistent with the detoxification function of F-cells (James and Boyle, 1998; Brouwer and Lee, 2007; Brignac-Huber et al., 2016). Previous *in situ* hybridization analyses have also implicated F-cells in immune defense (Wang et al., 2007). Here, several pathways related to viral infection and immune signaling were significantly enriched in the F-cells.

Some previous studies have suggested that B-cells secrete digestive enzymes, similar to F-cells (Barker and Gibson, 1979; Dall et al., 1983), while other studies have indicated that only F-cells have this function; indeed, immunohistochemical and *in situ* hybridization studies have shown that B-cells cannot synthesize classic digestive enzymes (Vogt et al., 1989). In addition, B-cells participate in the absorption of nutrients from the hepatopancreatic duct and concentrate absorbed substances in large vacuoles, but do not store nutrients (Al-Mohanna and Nott, 1986; Hopkin and Nott, 2009). E-cells are undifferentiated embryonic cells that can differentiate into R-cells, F-cells, and B-cells (Al-Mohanna and Nott, 1989; Toullec et al., 1992; Sousa and Petriella, 2000).

### Several Signaling Pathways Related to Cold Tolerance Were Enriched in *L. Vannamei*

Several pathways, including “oxidative phosphorylation,” “metabolism of xenobiology by cytochrome P450,” “chemical carcinogenesis,” “drug metabolism cytochrome P450,” and “fatty acid metabolism,” were significantly enriched in the cold-tolerant shrimp. Previous studies suggest that these signaling pathways may be important for cold tolerance. For example, oxidative phosphorylation provides ATP to meet increased energy demands under cold stress (Hall et al., 2012; Colinet et al., 2017; Chamberlain and Sheng., 2019), cytochrome P450 participates in detoxification and removal of harmful substances (Nanji and Hiller-Sturmhöfel., 1997; Nebert et al., 2004), and the fatty acid metabolism generates heat to cope with the low-temperature environments (Hui et al., 2014; Lee et al., 2019). *Drosophila melanogaster* uses the ATP produced by oxidative phosphorylation to meet energy requirements under cold stress (Williams et al., 2018). Indeed, it has been shown that cold tolerance in *L. vannamei* is associated with oxidative phosphorylation (Fan et al., 2019; Peng et al., 2018). Cytochrome P450 may be upregulated in shrimp in response to cold stress; cytochrome P450 may also participate in detoxification and the removal of harmful substances (Vergara-Amado et al., 2017), as well as the biotransformation of endogenous chemical products (such as reactive oxygen species) produced under chemical stress (Andersson and Förlin, 1992; Zhang et al., 2012). Increased cytochrome P450 expression has been detected in marine oligochaetes (*Thalassodrilides* sp.) exposed to pollutants and heat stress (Ito et al., 2016). Some functional analyses have shown that cytochrome P450 genes closely related to the fat metabolism (e.g., CYP7A1 and CYP1A1) play important roles in cold stress (Chen et al., 2012). Interestingly, exposure to low temperatures inhibits gene expression and most cellular processes in rats, with the exception of cytochrome P450 enzymes (CYP1A) in liver, which are instead activated by cold (Perepechaeva and Grishanova, 2013). Here, detoxification-associated genes were upregulated at 28°C or 10°C in the cold-tolerant shrimp (LV-T) as compared to the common shrimp (LV-C; Table 1). However, these genes were downregulated in response to low temperatures in both common and cold-tolerant shrimp (Table 1). This suggested that cold-tolerant shrimp may have greater detoxification capacity than the common shrimp, but that detoxification capacity in both lines was decreased by low-temperature stress.

The fatty acid metabolism is critical for cold adaptation (Gombos et al., 1994; Tuo et al., 2014; Zhang et al., 2020). Long-chain acyl-coenzyme A synthetase participates in the first step of the fatty acid metabolism and is one of the key enzymes for fat synthesis and catabolism (Wang et al., 2004). Studies have shown that the activity levels of long-chain acyl-CoA synthase increase in the rainbow trout heart during low temperature adaptation (Hicks et al., 1996; Patey and Driedzic, 2011), while fatty acyl-CoA synthetase-1 (adipose acyl-CoA synthetase-1, ACSL1) guides fatty acids to beta-oxidation, which is necessary for cold and heat generation (Ellis et al., 2010). In addition, stearoyl-CoA desaturase (SCD) is involved in the adaptation of large yellow croaker (*Pseudosciaena crocea*) to cold stress (Xu et al., 2015). Here, several genes associated with the fatty acid metabolism, including acyl-CoA dehydrogenase, long-chain-acyl-CoA dehydrogenase, and stearoyl-CoA desaturase, were significantly enriched in the cold-tolerant shrimp, indicating that the fatty acid metabolism plays an important role in the cold tolerance of *L. vannamei*. This was consistent with the results of Fan et al. (2019) and He et al. (2018).

Several immune-related genes, including C-type lectin (CTL), leukocyte receptor cluster member 8 (Lr8), hemocyanin (HC), integrin beta-PS-like (ITGB) and macrophage mannose receptor 1-like (MMR1), were upregulated in the cold-tolerant shrimp as compared to the common shrimp at 28°C or 10°C. In addition, CTL, HC, and MMR1 were upregulated in response to low-temperature stress, suggesting that the immune defense ability of the cold-tolerant shrimp was greater than that of the common shrimp. We speculate that low-temperature stress might affect the expression of immune-related genes, causing immune disorders and damaging the immune system.

Catalase (CAT), glutathione S-transferase (GST), glutathione peroxidase (GPX), and peroxiredoxin (PRDX4) are important antioxidants that can reduce ROS and prevent oxidative damage (Lim et al., 2018). GST, GPX, and PRDX4 were upregulated in cold-tolerant shrimp as compared to common shrimp at 28°C, suggesting that the immune defense abilities of cold-tolerant shrimp were better than those of common shrimp. In both cold-tolerant and common shrimp, these genes were downregulated in response to cold stress, indicating that low temperatures are associated with antioxidant system dysfunction in shrimp.

At both 10°C and 28°C, the taurine transporter (TAUT) was upregulated in the cold-tolerant shrimp as compared to the common shrimp. Taurine is an abundant, sulfur-rich free amino acid that is associated with antioxidant activity, ion transport, and DNA repair (Kuz’mina et al., 2010). Thus, our results suggested that the self-repair abilities of the cold-tolerant shrimp exceeded those of the common shrimp. In both common and cold-tolerant shrimp, heat shock protein 70 (HSC70) was upregulated in response to low temperature stress, suggesting that this protein is involved in the cold stress response mechanism. This was consistent with the results of Fan et al.(Fan et al., 2019).

### Reconstruction of F-Cell Differentiation Fate Trajectory Under Low Temperature Stress

F-cells participate in cell detoxification, immune defense, and the secretion of digestive enzymes. However, the differentiation trajectory of F-cells under low temperature stress is not clear. Reconstruction of the differentiation trajectory of F-cells will help to clarify the transcription factors that cause F-cell differentiation in response to cold stress. In response to low temperature stress, CP4, CPB, CTL, PTL, VMO1, and Zinc were upregulated over pseudotime in all cells (Figure 6A). CPB, a an immune-related metallopeptidase (Qian et al., 2019), requires zinc ions (Zn2+) to participate in catalysis; CPB has a variety of functions, including promoting protein digestion and the processing and formation hormones and neuropeptides. Zinc also plays an important role in low temperature stress in *Arabidopsis* (Nakai et al., 2013) and *L. vannamei* (Peng et al., 2018). Overexpression of Cystatin B increased resistance to salinity, drought, oxidative stress, and cold stress in transgenic yeast and *Arabidopsis* (Zhang et al., 2008). CTL is an important immune factor, and studies have suggested that *Osmerus mordax* CTL is homologous to antifreeze proteins (AFPs) (Achenbach and Ewart, 2002). Low temperature induces CTL expression in *Ruditapes philippinarum*. In squirrels (*Spermophilus tridecemlineatus*), PTL is strongly upregulated and has high activity levels at low temperatures; PTL provides a mechanism to release the fatty acids required by this species during hibernation (Squire et al., 2003).

CYP450, HC, and V-ATPase were downregulated in response to cold stress in all cells over pseudotime (Figure 6B). Fan et al. (2013) and Fan et al. (2019) found that, in response to cold stress, shrimp blood cell concentrations decreased and differentially expressed genes associated with hematopoiesis were downregulated. Here, we speculated that the shrimp hematopoietic system was damaged by exposure to cold, leading to the downregulation of HC. In crickets (*Gryllus pennsylvanicus*), cold acclimation can reduce the production of primary urine by downregulating carbonic anhydrase and V-ATPase in Markov tubules, thereby reducing the loss of hemolymph volume in cold environments (Des Marteaux et al., 2017). AST and VMO1 participate in the adaptive response of *Daphnia pulex* to environmental temperature changes (Schwerin et al., 2009). LDLR is an important regulator of cell differentiation and proliferation (Tsukamoto et al., 2012; Auderset et al., 2020).

After cold exposure, AST, Chia, CPB, CTL, LDLR2, MMR1, and Trypsin-1 were upregulated in the cell fate 2 branch (primarily associated with cold-tolerant shrimp cells) and slightly downregulated in the cell fate 1 branch (primarily associated with common shrimp cells); the converse relationship was observed for ALF, CLCA1, and HECA (Figure 6B). These genes determine the fate of F-cells under low temperature stress. Thus, the differences in gene expression dynamics between cold-tolerant and common cells may help to identify the transcription factors that drive differences in F-cell differentiation in response to low-temperature stress.

## Data Availability

The datasets presented in this study can be found in online repositories. The names of the repository/repositories and accession number(s) can be found below: https://www.ncbi.nlm.nih.gov/genbank/, SRP330719.
