# Supplementary material for: Single-Cell Ribonucleic Acid Sequencing Clarifies Cold Tolerance Mechanisms in the Pacific White Shrimp (Litopenaeus Vannamei)
Source: Front Genet. 2022 Jan 12;12:792172. doi: 10.3389/fgene.2021.792172 (PMC8790290; doi:10.3389/fgene.2021.792172)
Supplement: Supplementary file 1 [file Table1.DOCX]

**Additional Table 1.** Biological characteristics of cold-tolerant *Litopenaeus vannamei* (Lv-T) and common *Litopenaeus vannamei* (Lv-C)

| Biological characteristics | Lv-T | Lv-C |
| --- | --- | --- |
| Origin | obtained after 7 generations of breeding | a strain from Hawaii, USA |
| Average survival time at 10℃ | 30.07 hour | 20.17 hour |
| Food intake capacity | At 17℃to 20℃, the food intake is reduced by 20% compared to normal temperature; at >25℃, the food intake is normal. | At 17℃ to 20℃, the food intake is reduced by more than 75% compared to normal temperature; at >25℃, the food intake is normal. |
| Vitality | At 17℃ to 20℃, the amount of activity decreases; at >22℃, the activity is normal and the reaction is quick. | At 17℃ to 20℃, the amount of activity is extremely reduced; at >22℃, the activity is normal and the reaction is quick. |
| Molting cycle | At 17℃ to 20℃, molting occurs once every 10 to 15 days; at 20℃ to 22℃, molting occurs every 7 to 10 days. | When the temperature is above 25°C, molting occurs every 7 to 10 days. |
| Adaptation to temperature changes | The adaptability to temperature changes is strong, and the food intake increases rapidly with temperature rise. | The adaptability to temperature changes is weak, and the food intake increases slowly with temperature rise. |
| Natural overwintering ability | It can survive the winter naturally in Guangxi. | In the winter in Guangxi, it needs to be warmed. |
| Seedling cultivation temperature | When the water temperature is 26℃ to 28℃, the time from nauplii to larvae is 13±1d. When the water temperature is 30℃ to 32℃, the time from nauplius to larvae is 10±1d. | When the water temperature is 26℃ to 28℃, the development of nauplius is blocked, and it is basically impossible to grow to larvae. When the water temperature is 30℃ to 32℃, the time from nauplius to larvae is 10±1d. |
| Larvae incubation temperature | When the temperature is lower than 27℃, the fertilized eggs develop normally. | When the temperature is lower than 29℃, the development speed of the fertilized eggs slow down, and when the temperature is lower than 27℃, the development of the fertilized eggs stop. |
